# Improved Diagnostic Accuracy of Ameloblastoma and Odontogenic Keratocyst on Cone-Beam CT by Artificial Intelligence

**DOI:** 10.3389/fonc.2021.793417

**Published:** 2022-01-27

**Authors:** Zi-Kang Chai, Liang Mao, Hua Chen, Ting-Guan Sun, Xue-Meng Shen, Juan Liu, Zhi-Jun Sun

**Affiliations:** ^1^ The State Key Laboratory Breeding Base of Basic Science of Stomatology (Hubei-MOST) & Key Laboratory of Oral Biomedicine, Ministry of Education, School and Hospital of Stomatology, Wuhan University, Wuhan, China; ^2^ Department of Oral Maxillofacial-Head Neck Oncology, School and Hospital of Stomatology, Wuhan University, Wuhan, China; ^3^ Institute of Artificial Intelligence, School of Computer Science, Wuhan University, Wuhan, China

**Keywords:** deep learning, convolutional neural network, Inception v3, ameloblastoma, odontogenic keratocyst, cone-beam CT

## Abstract

**Objective:**

The purpose of this study was to utilize a convolutional neural network (CNN) to make preoperative differential diagnoses between ameloblastoma (AME) and odontogenic keratocyst (OKC) on cone-beam CT (CBCT).

**Methods:**

The CBCT images of 178 AMEs and 172 OKCs were retrospectively retrieved from the Hospital of Stomatology, Wuhan University. The datasets were randomly split into a training dataset of 272 cases and a testing dataset of 78 cases. Slices comprising lesions were retained and then cropped to suitable patches for training. The Inception v3 deep learning algorithm was utilized, and its diagnostic performance was compared with that of oral and maxillofacial surgeons.

**Results:**

The sensitivity, specificity, accuracy, and F1 score were 87.2%, 82.1%, 84.6%, and 85.0%, respectively. Furthermore, the average scores of the same indexes for 7 senior oral and maxillofacial surgeons were 60.0%, 71.4%, 65.7%, and 63.6%, respectively, and those of 30 junior oral and maxillofacial surgeons were 63.9%, 53.2%, 58.5%, and 60.7%, respectively.

**Conclusion:**

The deep learning model was able to differentiate these two lesions with better diagnostic accuracy than clinical surgeons. The results indicate that the CNN may provide assistance for clinical diagnosis, especially for inexperienced surgeons.

## Introduction

Ameloblastoma (AME) and odontogenic keratocyst (OKC) are common radiolucent lesions of the jaws in oral and maxillofacial surgery (1, 2). Radiographic examinations are vital for patients with odontogenic lesions, notwithstanding that histopathological findings are the gold-standard diagnostic criteria (3, 4). However, because of the overlap of morphological characteristics in radiography, it is usually difficult to accurately distinguish these two diseases. Current treatment modalities for AME are wide local excision and immediate reconstruction, but OKC is generally treated with more conservative surgical methods, such as marsupialization and/or enucleation. Given that they have different treatment strategies, it is imperative to differentiate these conditions before surgery (5–8).

**Figure 1:**
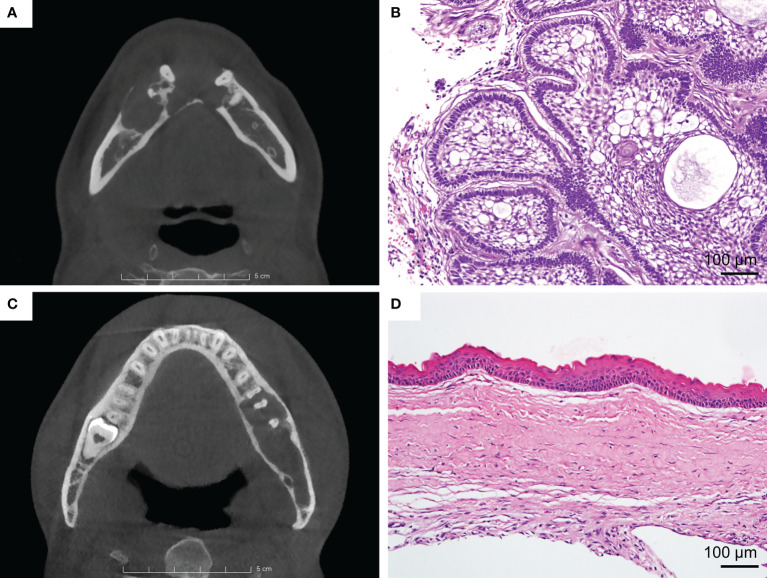
**(A)** Ameloblastoma (AME). Axial view of CBCT shows the lesion with buccal expansion, obvious cortical bone resorption, and a multilocular pattern. **(B)** Typical H&E staining of AME (×200). **(C)** Odontogenic keratocyst (OKC). Axial view of CBCT shows that the lesion grows along the bone, with unapparent disruption of the cortical bone and the unilocular pattern. **(D)** Typical H&E staining of OKC (×200).

Clinically, the differentiation between AME and OKC in radiography is mainly based on some features, such as buccolingual expansion, the number of locules, internal density, and the root resorption of the adjacent teeth (**Figure 1**). Nevertheless, only relying on these features is insufficient to obtain a strong differential diagnosis. Previous studies have sought more instrumental radiographic findings, such as the width-to-length ratio, volumetric measurement, and assessment of the Hounsfield unit, to distinguish these two lesions (9–11). However, these studies have the same limitation in that they only focused on low-level and limited features. Therefore, it can be contended that the current knowledge of radiography is still at tip of the iceberg, and more undetected information waits to be mined.

Recently, deep learning, which has been shown to outperform humans in object recognition and visual tasks, has achieved tremendous progress (12, 13). Deep learning algorithms have already been successfully used in medical practice, such as for the detection of incidental esophageal cancers, dermatologist-level classification of skin cancer, prediction of tyrosine kinase inhibitor treatment response, and diagnosis of COVID-19 pneumonia (14–17). In oral and maxillofacial oncology, some researchers have used deep learning methods to distinguish AME and OKC in panoramic radiographs and benefited greatly from the methods (18–20). However, panoramic radiography is not as good as cone-beam CT (CBCT) in demonstrating lesions. As the optimal examination for jaw lesions, CBCT has a high resolution, enabling it to comprehensively and clearly display lesions without distortion, superimposition, and misrepresentation of structures (21, 22). Lee et al. have demonstrated that their deep learning model trained with CBCT images performed better than that trained with panoramic images in diagnosing odontogenic cystic lesions (23). Consequently, we aimed to use a convolutional neural network (CNN) to automatically classify AME and OKC in CBCT data. Furthermore, we compared the diagnostic accuracy of the proposed model with that of senior and junior oral and maxillofacial surgeons.

## Materials and Methods

### Patient and Data Collection

The 350 patients in this study were obtained from the Hospital of Stomatology, Wuhan University, and all of them underwent surgical treatment with a diagnosis of jaw cystic disease from 2012 to 2020. The pathological diagnosis was made by one pathologist and reviewed by one pathologist from the Department of Oral Pathology, Wuhan University, based on criteria according to the World Health Organization Classification of Head and Neck Tumors (4th, 2017) (24). Their imaging data were retrieved from the picture archiving and communication system (PACS) and saved in DICOM format. All CBCT scans of patients were performed with the same CBCT device (NewTom VG, Italy). The tube voltage was set to 110 kV, and the tube current and the exposure time were regulated by the automatic exposure control system. The images were reconstructed with an isotropic voxel size of 0.3 mm and a 0.3-mm axial pitch.

The inclusion criteria were as follows: 1) complete clinical records, 2) definitive histopathological confirmation of the lesion as AME or OKC, and 3) availability of preoperative CBCT. The exclusion criteria included the following: 1) multiple OKCs or nevoid basal cell carcinoma syndrome and 2) images with apparent artifacts involving the regions of interest (ROIs).

Finally, an equalized dataset consisting of 178 AMEs (130 solid/multicystic ameloblastomas and 48 unicystic ameloblastomas) and 172 OKCs was included in this study. The data were randomly partitioned into two parts: 272 patients in the training set and 78 patients in the testing set, at a ratio of approximately 7:3 (**Table S1**).

### Image Processing

The CBCT data were loaded in the open source software 3D Slicer (version 4.11; www.slicer.org) and were demonstrated in three dimensions. The ROI of each slice was manually delineated by a junior surgeon using the semiautomatic segmentation method and then examined and modified by two professional surgeons. The labeled masks were saved in the axial sequence for the subsequent training process. To manifest the lesions more clearly, the open source software mDicom (MicroDicom) was utilized to adjust the raw DICOM images into the bone window (WW/WL, 1,000/300 HU), and then all axial sequences were exported as 512 * 512 pictures in PNG format.

**Figure 2:**
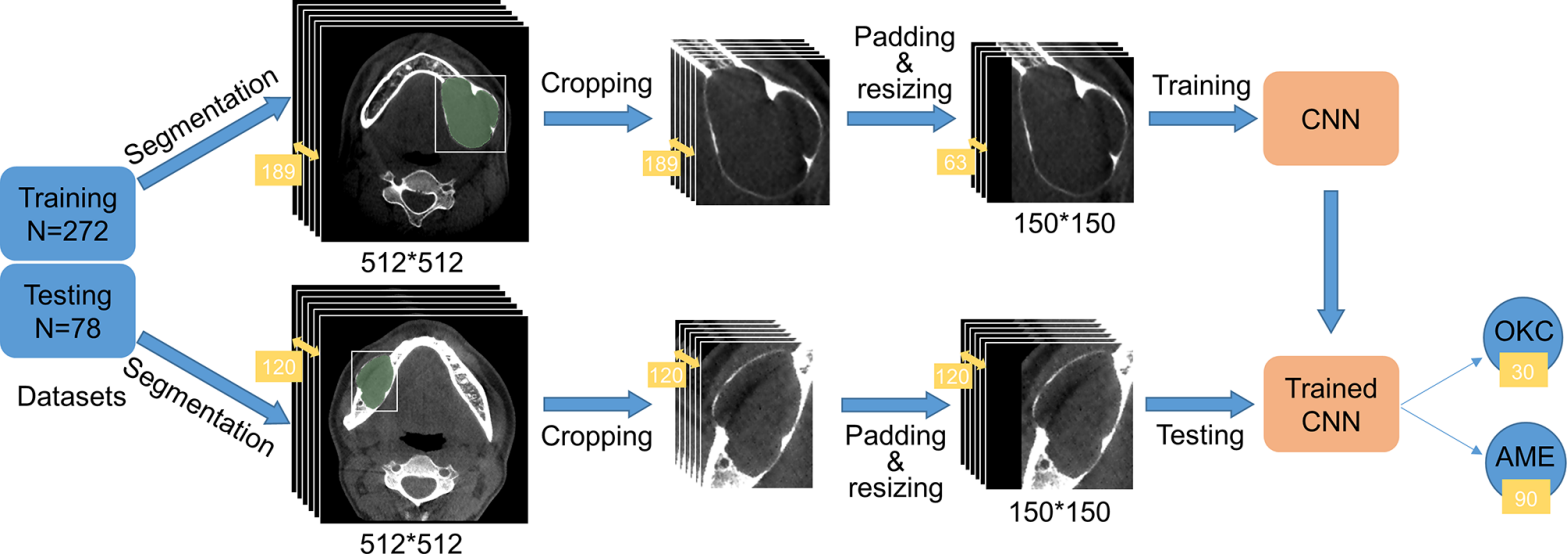
Flow diagram of the study. The training and testing datasets contained 272 and 78 patients, respectively. A total of 189 images comprising the region of interest (ROI) of one patient in the training dataset were cropped into smaller rectangles, padded into square images using black, and resized to 150 * 150 to gear the CNN. To reduce redundancy, we selected one image out of every three images. The series in the testing dataset underwent the same process except for changing the number of images. As shown in the picture, 120 slices of AME patient were tested by the trained CNN. Ninety slices were predicted to have AME and 30 slices were predicted to have OKC, so AME was ultimately considered.

**Figure 3:**
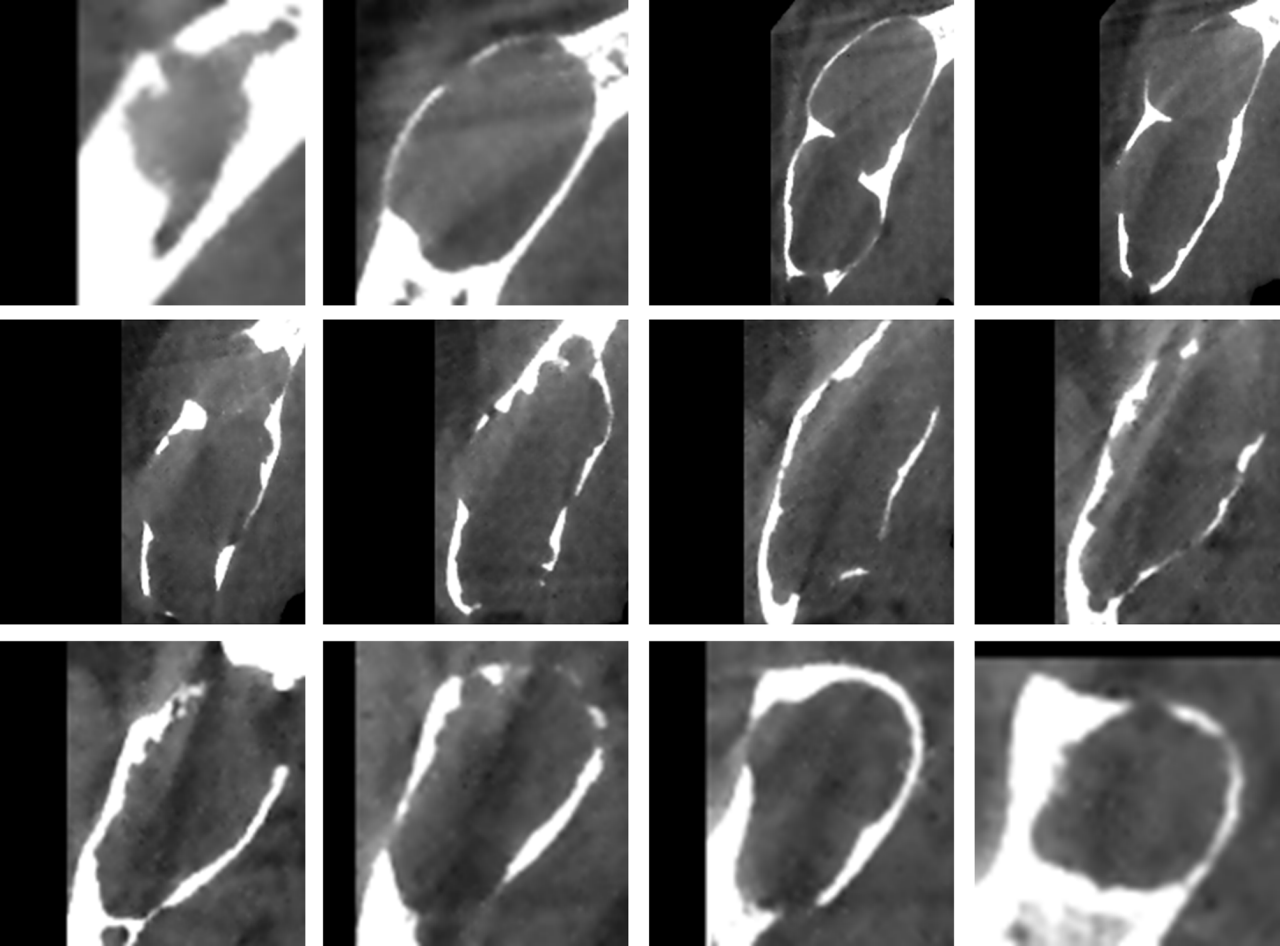
The images used for training were obtained after a series of processing steps, and these 12 images came from one patient.

The original pictures were cut into smaller rectangular patches that comprised only the lesions according to labeled masks. The rectangles should be reshaped to squares by padding the black-filled region to fit the CNN architectures and resized to 150 * 150 due to the inconsistent sizes of cropped images. In the training process, in order to reduce redundancy and avoid overfitting of the model, we selected one out of every three consecutive images in a series of each patient. Hence, only one-third of the images of each patient were retained. In the test phase, all images of each patient were tested, and the final classification result was up to the category with larger numbers. If the numbers of the two categories are equal, it means that the model made an incorrect diagnosis of the patient. The experimental procedure is illustrated in **Figure 2**, and some processed pictures of one patient are presented in **Figure 3**. After each case was processed identically, we obtained 272 patients in the training dataset and 78 in the testing dataset, consisting of 11,820 and 11,455 slices, respectively.

### Model Interpretation and Training Process

**Figure 4:**
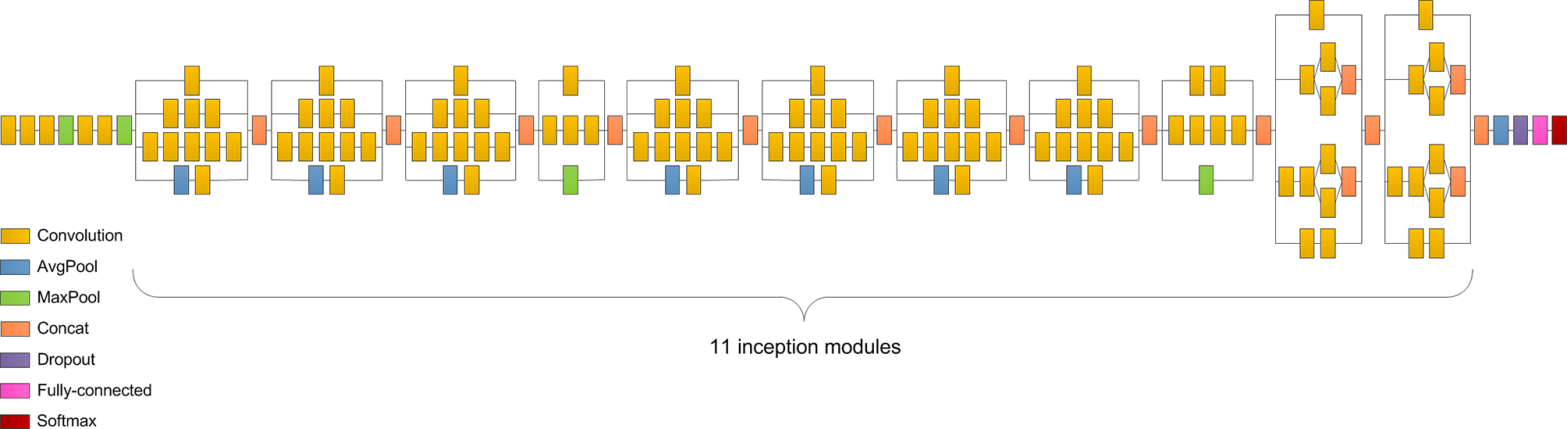
Inception v3 consists of five convolutional layers, two max-pooling layers, 11 inception modules, one average pooling layer, and one fully connected layer.

We selected the Inception v3 network as the classifier in our study because it performed better than the other three models (**Table S2**). Inception v3 clustered similar sparse nodes into a dense structure to increase both the depth and width of the network and reduce the computation process efficiently (25). The network consisted of five convolutional layers, two max-pooling layers, 11 inception modules, one average pooling layer, and one fully-connected layer (**Figure 4**). The convolution layers were used to extract the features in the jaw images. The pooling layers, including the max-pooling layers and average pooling layer, were utilized to reduce the dimension of features and reduce the amount of calculation. The inception module applied different sized convolution kernels to realize multiscale feature fusion. The fully connected layer integrated the output features of the convolution layer or pooling layer and output the probability value of each category after the Softmax activation function. To tackle the problem of limited dataset in medicine, transfer learning was applied in most situations. As done before (26), the CNN was trained on a large ImageNet dataset to learn the hierarchical features. Then, we applied the pretrained CNN with properly adjusted weights in our task.

**Figure 5:**
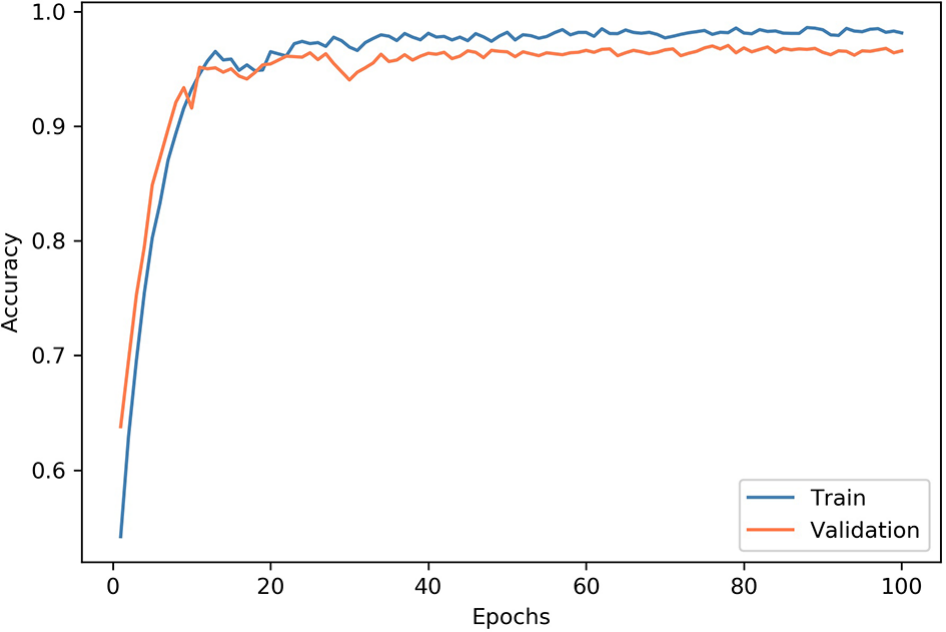
Convergence of the network training. At each epoch, the model was trained using all images in the training dataset, and the accuracy was evaluated. At the end of each epoch, we measured the accuracy of the model on the validation dataset. After 100 epochs, the training was stopped since both accuracy and cross-entropy loss would not be further improved.

In this work, our model was performed using a PC with the 64-bit Ubuntu 16.04 operating system, CUDA 9.0, an Intel E5-2650 v4 CPU, 256 GB RAM, a TITAN Xp GPU, and Python 3.5. In the model training process, the datasets were split into training and validation sets at a ratio of 4:1. We utilized the RAdam optimizer to train the layers in batches with a step size of 12 images and a learning rate of 0.0001. After 100 epochs, the training was stopped since both the accuracy and cross-entropy loss were not further improved. The learning history of the model is shown in **Figure 5**.

### Testing Surgeons

Clinical surgeons were tested using the identical testing dataset to obtain an objective assessment of the model. Seven senior surgeons and 30 junior surgeons participated in this study, and their results were classified into two groups: senior surgeons and junior surgeons. For each patient, two screenshots comprising three CBCT views, instead of the complete CBCT series, were offered for testing. Only the pictures of patients were summarized into a questionnaire with no more clinical information provided (**Figure S1**).

### Statistical Analysis


$$\text{Sensitivity}=\frac{\text{TP}}{\text{TP}+\text{FN}}$$

$$\text{Specificity}=\frac{\text{TN}}{\text{TN}+\text{FP}}$$

$$\text{Accuracy}=\frac{\text{TP}+\text{TN}}{\text{TP}+\text{TN}+\text{FP}+\text{FN}}$$

$$\text{F}1\;\text{score}=\frac{2*\text{TP}}{2*\text{TP}+\text{FP}+\text{FN}}$$

(TP: true positive, FP: false positive, TN: true negative, FN: false negative)

In this study, the accuracy, specificity, sensitivity (recall), and F1 score were used to assess the performance of Inception v3 and surgeons. For statistical analysis, we regarded the AME as positive and the OKC as negative. The sensitivity was derived by dividing the total number of patients correctly classified as having AME by the total number of AME cases. The specificity was derived by dividing the total number of patients correctly classified as having OKC by the total number of OKC cases. The accuracy was calculated by dividing the number of correctly classified patients by the total number of test patients. The F1 score is the harmonic average of the precision and recall and is considered to comprehensively measure classification performance.

## Results

### Patient Characteristics

The demographic and clinical data of the subjects in this study are presented in **Table 1**. The ages for AME cases range from 9 to 81 years, which is wider than the ages for OKC cases that range from 10 to 70 years. The average age of patients with AME and OKC are 40.3 ± 16.5 years (mean ± standard deviation) and 41.5 ± 17.6 years (mean ± standard deviation), respectively. Both AME and OKC have a predilection for the mandible.

**Table 1:** Demographic data of the study subjects.

Characteristics	OKC (*N* = 172)	AME (*N* = 178)
Age (mean ± SD)	41.5 ± 17.6	40.3 ± 16.5
Location		
Maxilla	63 (36.6%)	24 (13.5%)
Mandible	109 (63.4%)	154 (86.5%)
Gender		
Male	91 (52.9%)	108 (60.7%)
Female	81 (47.1%)	70 (39.3%)

SD, standard deviation; OKC, odontogenic keratocyst; AME, ameloblastoma.

### Comparison Results Between Model and Surgeons

Inception v3 obtained the highest scores among the participants, with a sensitivity of 87.4%, a specificity of 82.1%, an accuracy of 84.6%, and an F1 score of 85.0% (**Table 2**). For Inception v3, the diagnostic accuracy of AME (87.4%) was slightly higher than that of OKC (82.1%). Compared with lesions in the maxilla, the model had better diagnostic performance for the mandible, and the accuracies are shown in **Table 3**. The average prediction time for an image was 3.13 ms using the model, and the total time for diagnosing the 78 patients was 35.87 s.

**Table 2:** Comparison results of Inception v3 and surgeons.

	Sensitive (%)	Specificity (%)	Accuracy (%)	F1 score (%)
Inception v3	87.2	82.1	84.6	85.0
Senior surgeons	60.0	71.4	65.7	63.6
Junior surgeons	63.9	53.2	58.5	60.7

**Table 3:** Diagnostic accuracy in the maxilla and mandible.

	Testing number	Sensitive (%)	Specificity (%)	Accuracy (%)	F1 score (%)
Maxilla	15	50.0	81.8	73.3	50.0
Mandible	63	91.4	82.1	87.3	88.9

**Figure 6:**
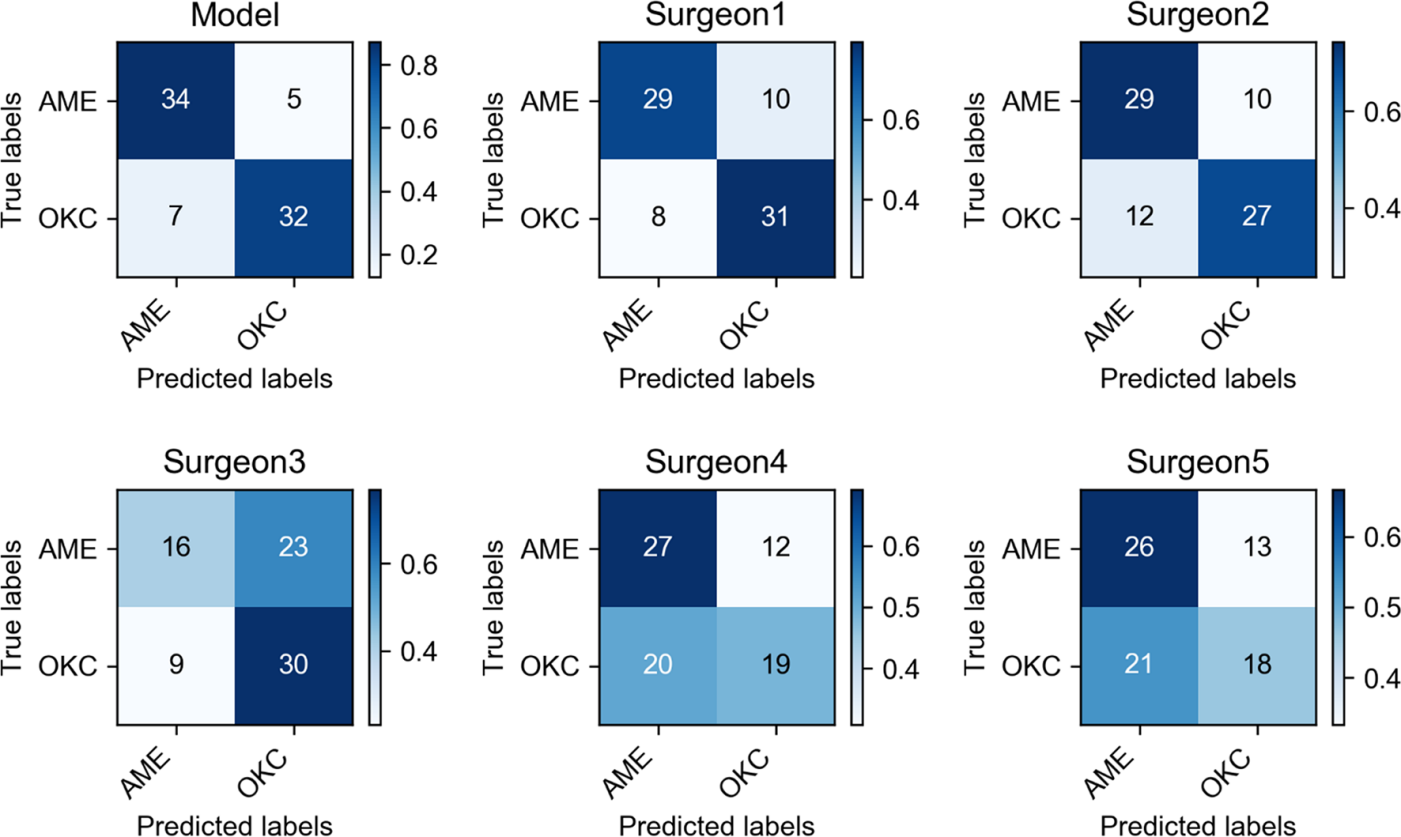
Confusion matrices of Inception v3 and five oral and maxillofacial surgeons showed the specific diagnostic performance. The color shade of the grid represented the proportion of each class.

The average sensitivity, specificity, accuracy, and F1 score for the classification of the group of 7 senior surgeons were 60.0%, 71.4%, 65.7%, and 63.6%, respectively, and those of the group of 30 junior surgeons were 63.9%, 53.2%, 58.5%, and 60.7%, respectively. The diagnostic outcomes of the CNN model and 5 surgeons were presented by confusion matrices (**Figure 6**). The average time to make diagnoses for 78 patients by 7 senior surgeons was 1,471 s. For the 30 junior surgeons, the average time was 1,113 s.

## Discussion

AME is the most common benign odontogenic tumor, accounting for approximately 10% of all odontogenic tumors (27). AME can arise from any odontogenic epithelium, so it can manifest widely varied radiographic findings. As the third most common odontogenic cyst, OKC represents nearly 12% of all odontogenic cysts, also arising from odontogenic epithelium (28). According to the literature reports, OKC is inclined to grow along the bone without the same buccolingual expansion of AME that usually results in bone resorption. However, these results could also be observed when OKC reached a large size. These confusing radiographic manifestations contributed to the difficulty of differential diagnosis. For AME, the main treatment modality is wide local excision and immediate reconstruction (6). Nevertheless, OKC is generally treated with more conservative surgical methods, such as marsupialization and/or enucleation, followed by adjunctive treatments, including cryotherapy with liquid nitrogen or the application of fixative Carnoy’s solution, to reduce recurrence (5). As a consequence, precise preoperative diagnosis is necessary for determining appropriate treatment strategies.

This study can be regarded as a successful application of deep learning in the field of odontogenic diseases with CBCT data. The results showed that our CNN model exhibited superior performance in differentiating AME and OKC compared with the oral and maxillofacial surgeons. Its diagnostic capability considerably outperformed senior and junior surgeons. Notably, though the sensitivity of junior surgeons (63.9%) was higher than that of the senior surgeons (60.0%), it did not mean that the junior surgeons had better diagnostic capabilities. This was because the junior surgeons in this study were inclined to choose the AME. As shown in the results, the specificity of junior surgeons (53.2%) was significantly lower than that of senior surgeons (71.4%). Furthermore, the CNN model spent extremely less time in diagnosis than the senior and junior surgeons. The average diagnosis time for the group of senior surgeons was longer than that for the group of junior surgeons. A possible explanation for this might be that senior surgeons would consider more details when they made a diagnosis. There are also some studies that developed deep learning models for differentiating AME and OKC in panoramic radiographs and achieved a high classification accuracy for lesions of the mandible (18–20). However, these models cannot perform well for lesions of the maxilla due to the inherent limitations of the panoramic radiograph, including the distortion, superimposition, and misrepresentation of structures. In contrast, CBCT has a higher resolution, enabling it to comprehensively and clearly display lesions in the maxillofacial region, which has many complex anatomic structures (21, 22). As a result, in our work, it was not necessary to deliberately select the location of onset. Our CNN model could substantially distinguish OKC and AME regardless of whether the lesion was in the maxilla or mandible. Bispo et al. used deep learning methods to differentiate them in multidetector CT images. However, their work was based only on extremely limited data from 40 patients, which would weaken the credibility of their results (29). In contrast, a larger dataset consisting of 350 patients was used in our study. Consequently, the convincing results indicated that our model could provide assistance for clinical diagnosis, especially for inexperienced surgeons.

In our study, we found that the diagnostic accuracy in the maxilla was lower than that in the mandible, and the possible explanations might be as follows. First, the low incidence in the maxilla results in less available data. Second, there may be more similar manifestations in the maxilla. There are few bone absorptions when lesions are small because of the intrinsic sinus cavities in the maxilla. However, the flimsy maxillary cortex is more susceptible to extensive destruction which often involves the nasal cavity and ethmoidal and sphenoidal sinuses, by both AME and OKC (30).

In the present study, two special and effective methods were used to improve the performance of the model. First, we cropped the original images using a tailored processing method. The original images contained many irrelevant anatomical structures, such as teeth, craniofacial bones, and muscles, and such loud noise might interfere with the model accurately extracting the features from the ROIs. Shin et al. proved that slice-level classification is more challenging than patch-level classification (31). Monkam and his colleagues compared the performances of several models based on different sized patches (32). Given that the sizes of ROIs in our database covered a large variation, it was irrational to establish a one-size-fits-all patch size. We tailored the optimal patch size for each slice by automatically measuring the mask to determine a suitable width and length of the rectangle. This process proved to be conducive to reducing the memory footprint and increasing the accuracy. Second, we noticed that the adjacent slices in the CBCT scans of one patient were extremely similar, which could lead to redundancy. As a solution, we selected one image out of every three images. This processing not only improved the training speed in every epoch but also effectively avoided overfitting and improved the model performance.

Keep in mind that our study still has some limitations. First, the diagnostic accuracy of the surgeons might be underestimated. Neither the model nor the surgeons were allowed to utilize the clinical information of patients, which is indispensable in clinical practice. In addition, we tested surgeons using only partial images of the CBCT series. Second, we did not perform external data validation; therefore, the generalizability of the model should be considered. The difficulty of obtaining sufficient images restricts the application of deep learning in the field of medical research. It is no exception that we used a relatively small amount of data, and all data were from the same medical center. Third, the CNN model was only based on 2D ROI patches of axial images, which might result in ignoring contextual information. Apparently, we suboptimally used the CBCT data, which are amenable to providing 3D manifestations. Ciompi et al. effectively classified pulmonary perifissural nodules by combining several 2D views (33), and Xu et al. designed a 3D CNN for automatic bladder segmentation to fully exploit 3D CT images (34). These studies of predecessors are bound to guide subsequent works, which are worthy of undertaking in the future. For example, we can attempt to multistream architectures based on three dimensions of CBCT or utilize a 3D-CNN to improve the classification accuracy. We can also search for the most suitable window setting to fully manifest lesions and pay more attention to overcoming the conundrum in differentiating lesions in the maxilla. Furthermore, external validations are indispensable to strengthen the generalization and credibility of the model. Additionally, we expect that deep learning will make greater advances and yield greater benefits for medical systems.

In conclusion, the CNN model achieved a fulfilling accuracy in diagnosing AME and OKC through CBCT, and the model significantly outperformed senior and junior surgeons of oral and maxillofacial. While these results require further validation, our work suggests that the CNN model can provide substantial assistance with non-invasive diagnosis and therapy guidance for patients.

## Data Availability Statement

The datasets presented in this article are not readily available because we want to protect the interest of the patients. Requests to access the datasets should be directed to sunzj@whu.edu.cn.

## Ethics Statement

This study followed the Declaration of Helsinki guidelines and its protocol was approved by the Institutional Medical Ethics Committee of School and Hospital of Stomatology, Wuhan University (2018LUNSHENZIA28). Written informed consent to participate in this study was provided by the legal guardian/next of kin of the participants. Written informed consent was obtained from the legal guardian/next of kin of the individual(s) and minor(s), for the publication of any potentially identifiable images or data included in this article.

## Author Contributions

Z-JS and JL: study conception and design. Z-KC, T-GS, and X-MS: data collection. HC and Z-KC: data analysis and interpretation. Z-KC and LM: manuscript writing. Z-JS and JL: manuscript revision. All authors: manuscript review and final approval of the manuscript.

## Funding

This work was supported by the National Natural Science Foundation of China (82072996 and 82103333), the Fundamental Research Funds for the Central Universities (2042021kf0216), the Major Projects of Technological Innovation in Hubei Province (2019AEA170), and the Frontier Projects of Wuhan for Application Foundation (2019010701011381).

## Conflict of Interest

The authors declare that the research was conducted in the absence of any commercial or financial relationships that could be construed as a potential conflict of interest.

## Publisher’s Note

All claims expressed in this article are solely those of the authors and do not necessarily represent those of their affiliated organizations, or those of the publisher, the editors and the reviewers. Any product that may be evaluated in this article, or claim that may be made by its manufacturer, is not guaranteed or endorsed by the publisher.
