# Supplementary material for: Improved Diagnostic Accuracy of Ameloblastoma and Odontogenic Keratocyst on Cone-Beam CT by Artificial Intelligence
Source: Front Oncol. 2022 Jan 27;11:793417. doi: 10.3389/fonc.2021.793417 (PMC8828501; doi:10.3389/fonc.2021.793417)
Supplement: Supplementary file 1 [file Image_1.pdf]

Supplementary Material

1.1 Supplementary Figures

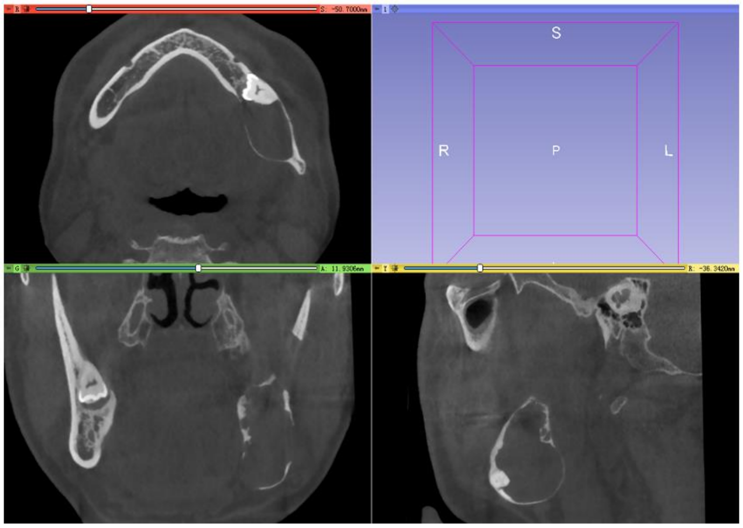

Supplementary Figure 1. Picture comprising three views of CBCT were offered for testing surgeons.
