# Supplementary material for: Improved Diagnostic Accuracy of Ameloblastoma and Odontogenic Keratocyst on Cone-Beam CT by Artificial Intelligence
Source: Front Oncol. 2022 Jan 27;11:793417. doi: 10.3389/fonc.2021.793417 (PMC8828501; doi:10.3389/fonc.2021.793417)
Supplement: Supplementary file 2 [file Table_1.docx]

Supplementary Material

**Supplementary Table 1 Patients in training and testing sets**

|  | AME | |  | OKC | |
| --- | --- | --- | --- | --- | --- |
|  | Maxilla | Mandible |  | Maxilla | Mandible |
| Training set | 20 | 119 |  | 52 | 81 |
| Testing set | 4 | 35 |  | 11 | 28 |

**Supplementary Table 2 Classification performance of different CNNs**

|  | Sensitive(%) | Specificity(%) | Accuracy(%) | F1score(%) |
| --- | --- | --- | --- | --- |
| Inception v3 | 87.2 | 82.1 | 84.6 | 85.0 |
| VGG16 | 84.6 | 74.4 | 79.5 | 80.5 |
| ResNet50 | 84.6 | 76.9 | 80.8 | 81.5 |
| DenseNet121 | 87.2 | 74.4 | 80.8 | 81.9 |
